# Predictive value of neutrophil-to-lymphocyte ratio in diagnosis of prostate cancer among men who underwent template-guided prostate biopsy: A STROBE-compliant study

**DOI:** 10.1097/MD.0000000000005307

**Published:** 2016-11-04

**Authors:** Tian-bao Huang, Shi-yu Mao, Sheng-ming Lu, Jun-jie Yu, Yang Luan, Xiao Gu, Hao Liu, Guang-chen Zhou, Xue-fei Ding

**Affiliations:** a Department of Urology, Northern Jiangsu People's Hospital, Yangzhou, Jiangsu Province, China; b Department of Urology, College of Clinical Medicine, Yangzhou, Jiangsu Province, China; c Department of General Surgery, Northern Jiangsu People's Hospital, Yangzhou University, Yangzhou, Jiangsu Province, China; d Department of Urology, Shanghai Tenth People's Hospital, Shanghai, China.

**Keywords:** neutrophil-to-lymphocyte ratio, prostate biopsy, prostate cancer, retrospective study

## Abstract

To evaluate the predictive value of neutrophil-to-lymphocyte ratio (NLR) in diagnosis of prostate cancer (PCa). Data of 662 patients who underwent prostate biopsy from January 2012 to June 2016 were retrospectively reviewed. The receiver operating characteristic–derived area under the curve analyses were performed to assess the predictive accuracy. Simultaneously, Youden's index was calculated to determine the optimal NLR cutoff. Furthermore, univariate and multivariate logistic regression analyses were performed to determine the association between NLR value and PCa detection. On account of an NLR value of 2.44 was shown with the maximal Youden's index on the receiver operating characteristic curve, the cutoff value of NLR was set at 2.44. Accordingly, patients were classified into high-NLR or low-NLR group. The patients in high-NLR group might have significant higher risk to be diagnosed with PCa (HR 1.640; *P* = 0.031), especially in the subgroup with prostate-specific antigen (PSA) ranged from 4 to 10 ng mL^−1^ (hazard ratio [HR] 4.364; *P* = 0.003). The high-NLR was independent of age of diagnosis, PSA, prostate volume, abnormal digital rectal examination, and hypoechoic lesion on transrectal ultrasound for positive prostate biopsy. In the so-called gray area, combination of NLR value could raise 4.6% of the accuracy of the multivariate logistic model in PCa prediction, but not in advanced PCa prediction.

The patients with high-NLR value may have significant higher risk to be diagnosed with PCa, especially among the patients with PSA ranged from 4 to 10 ng mL^−1^. In this subgroup, the adding of NLR value in the multivariate model can improve the accuracy of PCa prediction in a large degree. If validated, the NLR will become a promising, accessible, inexpensive biomarker for PCa prediction.

## Abstract 2

Supplemental Digital Content is available in the text

## 1 Introduction

Prostate cancer (PCa) is the most commonly diagnosed non-skin cancer and the second leading cause of cancer death in man in United State.^[1]^ In China, approximate 60,300 new cancer cases and 26,600 cancer deaths related to PCa are projected to occur in 2015.^[2]^ Several factors, such as gradual implementation of prostate-specific antigen (PSA) screening, improved biopsy techniques and increasing westernized lifestyle, may partial explain this tendency.^[2]^

Nowadays, transrectal ultrasound (TRUS)-guided prostate biopsy is still the gold standard of diagnosis of PCa. PSA is a member of the kallikrein-related peptidase family, which has been widely used for early detection of PCa. Elevated PSA level is a most common indication for prostate biopsy. However, apart from enabling more definitive PCa diagnosis, PSA screening also results in a certain amount of unnecessary biopsy, especially among patients in the so-called gray area. Besides, approximately 1 out of 5 men with PCa might be misdiagnosed in the first prostate biopsy. Therefore, novel markers are needed, which can both detect clinically significant PCa and prevent unnecessary biopsies.

The relations between inflammation and cancer were disputed for a long time. The detailed mechanism was still not clear, but it was confirmed that the inflammation plays a critical role in carcinogenesis. Neutrophil-to-lymphocyte ratio (NLR) is one of the inflammatory parameters, which has been reported having prognostic value in some solid cancers, including PCa.^[3–7]^ Elevated NLR was closely associated with poor overall survival and progression-free survival/recurrence-free survival in patients with PCa, especially in patients with metastatic castration-resistant PCa.^[5]^ Several studies,^[8–12]^ trying to assess the predictive value of NLR in diagnosis of PCa, were published with controversial results. We, therefore, conducted this retrospective study of patients from January 2012 to June 2016 to further assess the potential association between NLR value and PCa diagnosis.

## 2 Material and methods

A total of 662 patients, who underwent transperineal TRUS-guided template-guided prostate biopsy in Northern Jiangsu People's Hospital from January 2012 to June 2016, were retrospectively reviewed with the permission of the ethical committee of the hospital. All data were analyzed anonymously. Puncture indications are as follows: abnormal findings in digital rectal examination (DRE) with any PSA value; abnormal signal in B ultrasound or computed tomography or magnetic resonance imaging examination; PSA more than 10 ng.ml^−1^ with any f/t PSA and PSA density; 4) PSA range from 4 to 10 ng.ml^−1^ with abnormal f/t PSA or PSA density value. In all patients, the prostate was routinely biopsied by using 11-region template. The detailed methods were described previously.^[13]^ Patients with symptomatic prostatitis or urinary tract infection or systemic inflammatory disease or any history of anti-inflammatory drug use within the 2 weeks before the biopsy were excluded. Besides, patients with high-grade prostatic intraepithelial neoplasia were also excluded. Finally, the patients, whose PSA before biopsy was less than 100 ng.ml^−1^ and imaging material indicated no metastasis, were included in the study.

For each patient, blood examination indexes such as white blood cell, neutrophil, lymphocyte, platelet count and prebiopsy PSA level, and clinical parameters like age of diagnosis, prostate volume, abnormal DRE (yes or no), hypoechoic lesion on TRUS (yes or no), number of biopsy cores, and biopsy results were collected. For patients who were diagnosed with PCa, Gleason score was also gathered. NLR was calculated by dividing the neutrophil count by the lymphocyte count, while platelet-to-lymphocyte ratio (PLR) was computed by dividing the platelet count by the lymphocyte count.

### 2.1 Statistical analysis

All the statistical analyses were performed using statistical software package SPSS for Windows, version 19. Patients were firstly grouped with regard to histology of the biopsy. Abnormal DRE and hypoechoic lesion on TRUS were shown as frequency (percentage) and compared by using chi-square test. All the blood indexes and other clinical parameters were displayed as the median (interquartile range [IQR]) and compared by using Mann–Whitney *U* test. Advanced PCa were defined as PCa with Gleason score ≥4+3. Then, the receiver operating characteristic (ROC)-derived area under the curve (AUC) analyses were performed to assess the predictive accuracy. Simultaneously, Youden's index was calculated to determine the optimal NLR cutoff. Furthermore, univariate and multivariate logistic regression analyses were performed to determine the association between NLR value and PCa detection. A 2-tailed *P* value of less than 0.05 was considered statistically significant.

## 3 Results

A total of 802 patients who underwent transperineal TRUS-guided template-guided prostate biopsy were recorded. Of these, 95 patients were excluded for prebiopsy PSA more than 100 ng.ml^−1^. Besides, 23 patients with inflammatory disease and 10 patients with history of anti-inflammatory drug use within the 2 weeks before the biopsy were excluded. Nine patients who diagnosed as high-grade prostatic intraepithelial neoplasia were also excluded. In addition, another three patients were excluded for having metastasis disease. Finally, 662 patients were included in present study.

**Figure 1:**
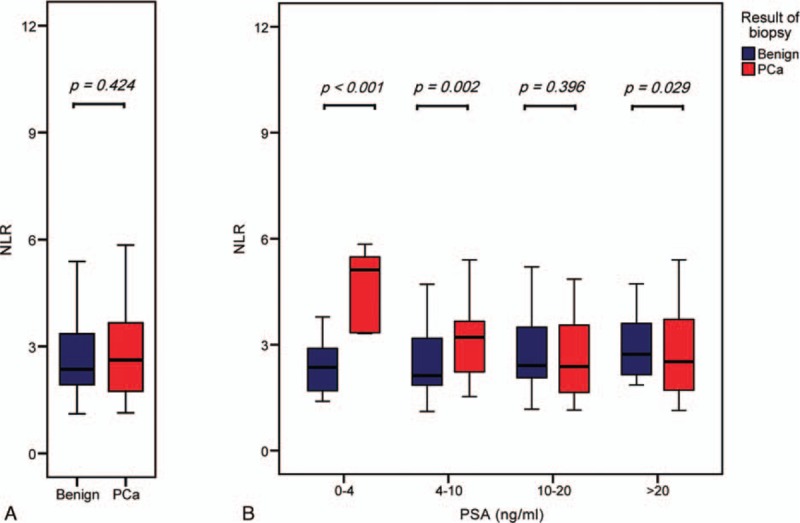
Box plot of NLR value grouped by pathologic results in prostate biopsy. (A) In the entire cohort. (B) In the 4 groups with different PSA range: 0 to 4, 4 to 10, 10 to 20, more than 20 ng.ml^−1^. There was no statistically significant difference between these 2 groups in the entire cohort (median 2.62, IQR 1.74–3.66; median 2.36, IQR 1.93–3.36, respectively, *P* = 0.424), but significant differences in the 3 subgroups except a subgroup with PSA ranged from 10 to 20 ng.ml^−1^. NLR = neutrophil-to-lymphocyte ratio, IQR = interquartile range, PCa = prostate cancer, PSA = prostate specific antigen.

The median age of the 662 men included in present study was 69 (IQR 63–75, range 40–90) years and the median PSA level was 14.32 (IQR 7.45–25.09) ng.ml^−1^ (Table 1). Among all the individuals, PCa was detected in 317 (47.9%), of which, 209 men were with high Gleason score (≥4+3). Patients were firstly grouped with regard to histology of the biopsy. Except for age (*P* < 0.001), PSA value (*P* < 0.001), prostate volume (*P* < 0.001), and platelet count (*P* = 0.017), we did not find any significant difference between groups in other parameters, included NLR value (*P* = 0.424) (Fig. 1A). However, we observed additional significant differences in lymphocyte count (*P* = 0.003) and NLR value (*P* = 0.002) between groups, when restricted our analyses to patients with PSA ranged from 4 to 10 ng.ml^−1^ (Table 2, Fig. 1B).

**Table 1:**
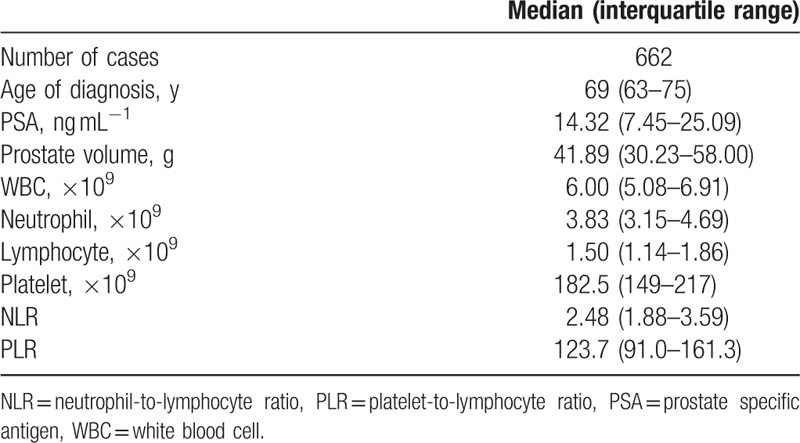
Clinical characteristics and blood parameters of the patients in the entire cohort (n = 662).

**Table 2:**
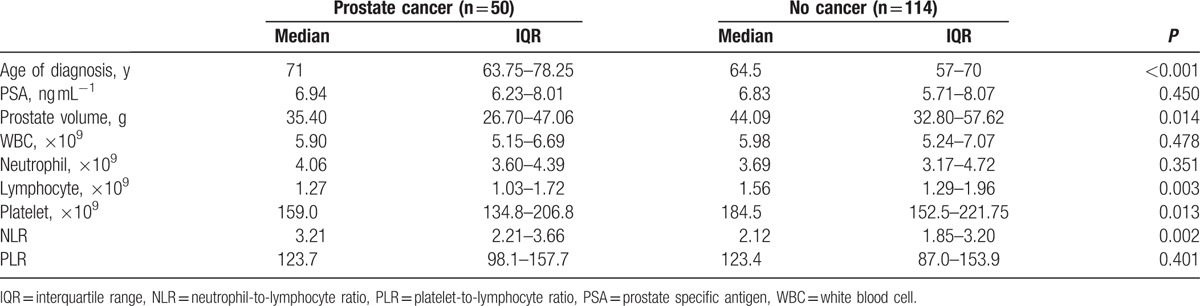
Clinical characteristics and blood parameters in patients with or without prostate cancer in the subgroup with PSA ranged from 4 to 10 ng.ml^−1^.

Then, we performed ROC analysis to assess the sensitivity and specificity in PCa prediction of the NLR value. As can be observed, in entire cohort, the AUC of NLR value was 0.518 (*P* = 0.424). Nevertheless, in subgroup with PSA ranged from 4 to 10 ng.ml^−1^, the AUC of NLR was 0.654 (*P* = 0.002). On account of an NLR value of 2.44 was shown with the maximal Youden's index on the ROC curve, the cutoff value of NLR was set at 2.44.

Accordingly, patients were classified into high-NLR or low-NLR group (detailed in Table 3). The distribution of age, PSA, prostate volume, abnormal DRE, and hypoechoic lesion on TRUS, but not Gleason score, were with significant differences between the 2 groups in the entire cohort. Besides, the high-NLR group showed significantly high PCa detection rate than the low-NLR group (175/338, 142/324, *P* = 0.041). Further analyses showed that the significance was retained among the patients with PSA ranged from 4 to 10 ng.ml^−1^ (36/77, 14/87, *P* < 0.001). Univariate logistic regression analysis showed that high-NLR was associated with high possibility to be diagnosed as PCa in the prostate biopsy (*P* = 0.041) (Table 4). Further multivariate analysis revealed that the high-NLR was independent of age of diagnosis, PSA, prostate volume, abnormal DRE and hypoechoic lesion on TRUS for positive prostate biopsy (hazard ratio [HR] 1.640; 95% confidence interval [CI] 1.045–2.573, *P* = 0.031). To our surprise, among patients with PSA ranged from 4 to 10 ng.ml^−1^, compared with low-NLR group, patients in high-NLR group had 4.364-fold risk to be diagnosed as PCa (*P* < 0.001) (Table 4). Apart from that, we did not find any significant association between NLR value and diagnosis of advanced PCa in either entire cohort (*P* = 0.258) or subgroup with PSA ranged from 4 to 10 ng.ml^−1^ (*P* = 0.075) in the multivariate analyses.

**Table 3:**
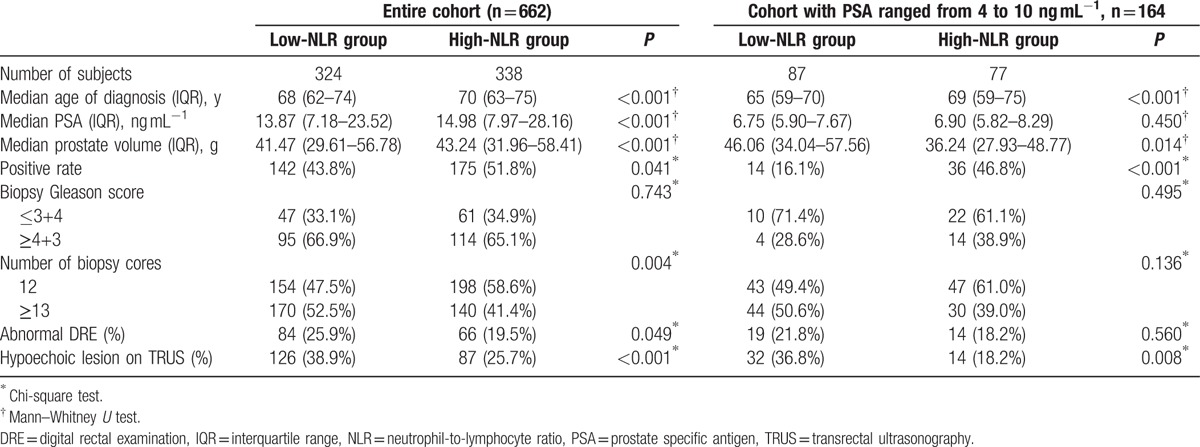
Clinicopathological characteristics of the entire cohort and cohort with PSA ranged from 4 to 10 ng.ml^−1^.

**Table 4:**
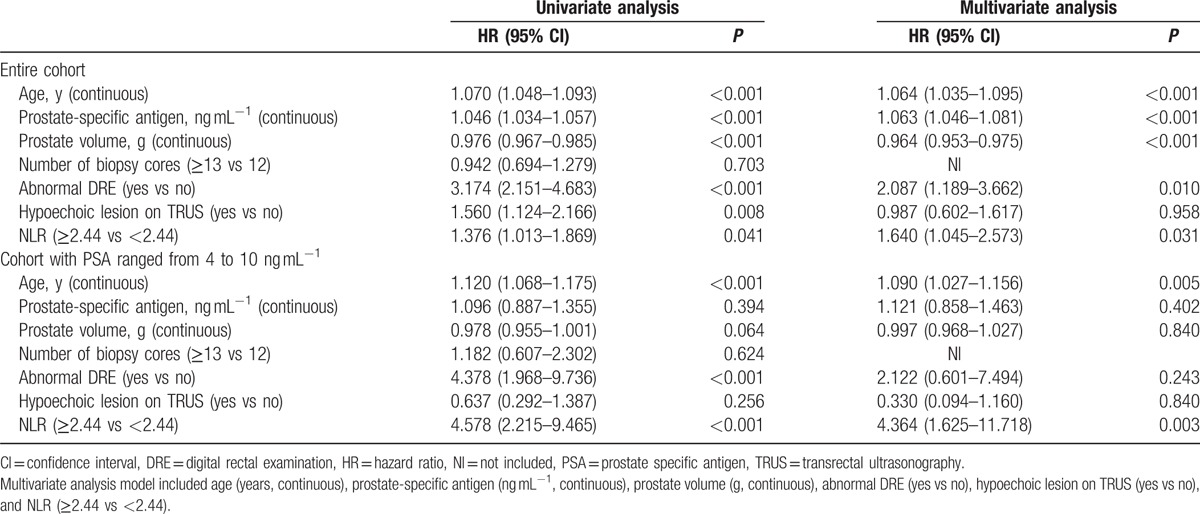
Univariate analysis and multivariate analyses of the impact of high neutrophil–lymphocyte ratio (NLR ≥2.44) on PCa detection.

**Figure 2:**
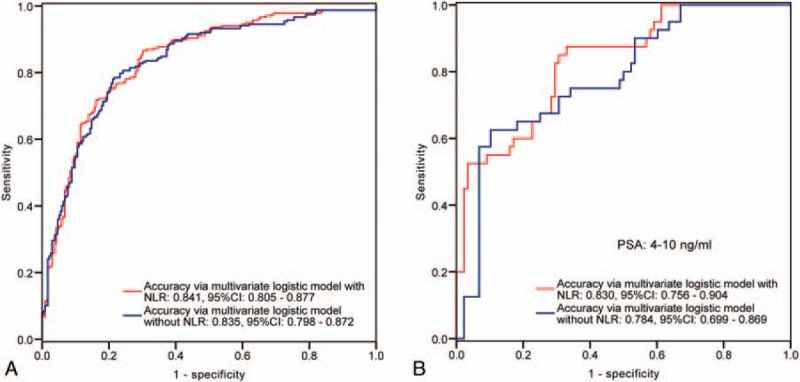
ROC curves of the multivariate logistic regression model with or without NLR value in prediction of PCa in the entire cohort (A) and in the subgroup with PSA ranged from 4 to 10 ng.ml^−1^ (B). As can be observed, in the entire cohort, the accuracy of the multivariate logistic model with NLR is close to the model without NLR. Nevertheless, in the subgroup with PSA ranged from 4 to 10 ng.ml^−1^, the adding of NLR value leads 4.6% up of the accuracy. AUC = area under the curve, NLR = neutrophil-to-lymphocyte ratio, PCa = prostate cancer, PSA = prostate specific antigen, ROC = receiver operating characteristic.

**Figure 3:**
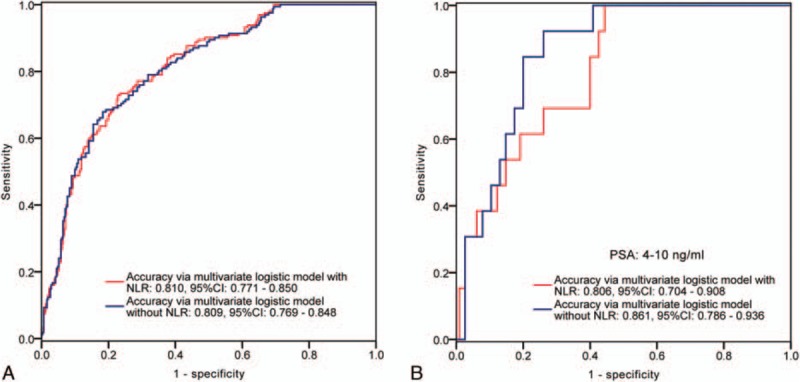
ROC curves of the multivariate logistic regression model with or without NLR value in prediction of advanced PCa (≥4+3) in the entire cohort (A) and in the subgroup with PSA ranged from 4 to 10 ng.ml^−1^ (B). As can be observed, in the entire cohort, the accuracy of the multivariate logistic model with NLR is close to the model without NLR. Nevertheless, in the subgroup with PSA ranged from 4 to 10 ng.ml^−1^, the adding of NLR value leads 5.5% down of the accuracy. AUC = area under the curve, NLR = neutrophil-to-lymphocyte ratio, PCa = prostate cancer, PSA = prostate specific antigen, ROC = receiver operating characteristic.

Finally, we performed ROC analyses to compare the predictive accuracy calculated from the multivariate logistic model with or without NLR value. In the entire cohort, the accuracy level changed little by adding NLR value into multivariate model in either prediction of PCa or prediction of advanced PCa (Figs. 2A and 3A). However, in subgroup with PSA ranged from 4 to 10 ng.ml^−1^, the accuracy level increased by 4.6% in prediction of PCa (AUC 0.830, AUC 0.784, respectively) (Fig. 2B), but decreased in prediction of advanced PCa (AUC 0.806, AUC 0.861, respectively) (Fig. 3B), when added the NLR value into the multivariate logistic model.

## 4 Discussion

Accumulating evidence showed that systemic inflammation was positively associated with various solid cancer types, including colorectal, gastric, and lung cancers.^[14–16]^ Markers,^[8,17–21]^ which have been commonly used, included C-reactive protein, neutrophil count, platelet count, prostate health index, modified Glasgow Prognostic Score, NLR, and PLR. Guthrie et al^[17]^ summarized that NLR was elevated in patients with more advanced or aggressive disease. By performing a systematic review of one hundred correlated studies, Templeton et al^[14]^ revealed that high NLR was associated with poor overall survival in many solid tumors (HR 1.81 95% CI 1.67–1.97). As to PCa, elevated C-reactive protein value was a strong predictor of poor survival and lower probability of PSA response to treatment in patient with metastatic castration-resistant PCa who were receiving docetaxel-based therapy.^[22]^ Besides, elevated NLR was closely associated with poor overall survival in patients with PCa.^[5]^ Another study in Japan,^[6]^ which was included 1464 patients, revealed that NLR was correlated with both cancer-specific survival (*P* = 0.018) and overall survival (*P* = 0.008) in patients with metastatic PCa.

Apart from prognostic value of inflammation on PCa, there were also several studies assessing inflammation markers on PCa risk. Bruzzese et al^[21]^ performed a meta-analysis by including 8 observational studies, and revealed that prostate health index is a promising predictor of positive first biopsy (AUC 0.74 95% CI 0.70–0.77). Men with elevated leukocyte count were associated with higher PCa risk (highest tertile vs lowest tertile, HR 1.60; *P* trend = 0.01) and higher PCa mortality (HR 2.57, 95% CI 0.99–6.79).^[23]^ While a study in Japan revealed that elevated neutrophil count was associated with higher possibility of a benign prostate biopsy.^[24]^ After that, several studies^[8–12]^ on the predictive value of NLR in diagnosis of PCa were published with controversial results. Yuksel et al^[8]^ reviewed 873 patients who underwent prostate biopsy and found that NLR value in PCa patients were similar with patients without PCa (mean 3.03; mean 3.04, respectively, *P* = 0.944). Study by Gokce et al,^[11]^ which including 1836 patients, also revealed a analogous result. Further analyses presented that it is prostatitis, which prevents the use of NLR in predicting PCa before prostate biopsy. However, in another 2 studies,^[9,10]^ significant differences were exhibited between patients with and without PCa (both *P* < 0.001). Multivariate analyses revealed that the higher NLR was independently associated with PCa detection (both *P* < 0.05). In present study, we observed an interesting phenomenon that NLR value had a poor predictive value in entire cohort without limiting PSA value, but a promising superior predictive value among patients with PSA ranged from 4 to 10 ng.ml^−1^. In this subgroup, compared with low-NLR group, patients in high-NLR group had 4.364-fold risk to be diagnosed as PCa (*P* < 0.001). Besides, the combination of NLR could raise the accuracy of multivariate model including age, PSA, prostate volume, abnormal DRE, hypoechoic lesion on TRUS in the subgroup with PSA ranged from 4 to 10 ng.ml^−1^ (AUC 0.830, AUC 0.784, respectively).

The connection between inflammation and cancer was initially hypothesized by Rudolf Virchow in 1863. He noticed that cancer originated at the site of chronic inflammation and was triggered by chronic inflammation.^[9,25]^ Thereafter, several studies were published and revealed that inflammatory cytokines networks had important impact on survival, proliferation and differentiation of tumor cells through DNA damage,^[26]^ angiogenesis,^[27,28]^ and some other signal transduction pathways.^[25,29]^ In addition, tumor cells coopted selectins, chemokines, and some other signaling molecules of the innate immune system for invasion, migration, and metastasis.^[30]^ So far, the molecular and cellular mechanisms between inflammation and cancers’ biological characteristics are still unresolved, but the relations have been widely accepted.

Several studies^[31–34]^ supported a role of prostatic inflammation in the etiology of PCa. Chronic inflammation was involved in prostate carcinogenesis through disrupting of the immune response and regulating of the tumor microenvironment.^[35]^ Besides, growing indirect evidences^[36–38]^ have emerged that nonsteroidal anti-inflammatory drugs, especially aspirin use might reduce the incidence in various cancers including PCa. Previously, we conducted a meta-analysis,^[39]^ which included 14 case–control studies and ten cohort studies, and revealed that regular aspirin use, especially long-term regular aspirin use might reduce the risk of PCa. Cyclooxygenase-2 enzyme which aberrantly expressed in PCa tissue^[40,41]^ might partially explain the potential association between aspirin use and PCa risk. Through cyclooxygenase-2–independent pathway, aspirin not only reduced the synthesis of prostaglandin,^[42]^ but also inhibited the cellular proliferation and angiogenesis by upregulating of tumor suppressor genes.^[39,43]^ Direct interactions of prostaglandins with their receptors through autocrine or paracrine pathways could enhance cellular survival and stimulate angiogenesis.^[44]^

However, Sciarra et al^[32]^ considered that it might be too early to use integrate inflammation in risk stratification analysis of prostate disease. Inflammation of prostate which was secondary to infection might differ from which arose from proliferative inflammatory atrophy lesion. The former may have a role in benign prostatic hyperplasia (BPH) progression, while the latter may increase the prostate's vulnerability to develop cancer. But inflammation markers, like NLR value, might elevate as long as inflammation exist. This might be a basal reason for insignificant association between the NLR value and diagnosis of PCa in the entire cohort. But in the subgroup, in which patients had a PSA ranged from 4 to 10 ng.ml^−1^, high-NLR was significantly associated with higher risk to be diagnosed with PCa. Previously, by comparing the distribution of NLR among patients with PCa, prostatitis and BPH, Gokce et al^[11]^ found that the presence of prostatitis might limit the usage of NLR in prediction of PCa. On this occasion, we hypothesis that among patients with relatively lower PSA level, the influence of prostatitis in the prediction value of NLR will become weak, due to lower PSA will get rid of several patients with prostatitis in a certain degree. More studies are needed to explain the discrepancy value of NLR in PCa prediction with different range of PSA value.

Recently, Yuksel et al^[8]^ conducted a study including 265 PCa and 304 BPH patients and revealed that PLR were significantly higher in the PCa group relative to BPH group (mean 134.4, standard deviation [SD] 76.2; mean 124.4, SD 76.2, respectively, *P* = 0.018). However, in present study, PCa patients exhibited similar PLR values to the patients without PCa either in entire cohort (median 119.1, IQR 89.4–158.4; median 126.8, IQR 92.1–162.4; *P* = 0.314) or in the subgroup (median 124.3, IQR 100.3–166.5; median 122.4, IQR 87.0–149.5; *P* = 0.098). The discrepancies were mainly due to the limitation of sample size and retrospective property. More prospective studies were needed to further assess this topic.

Several limitations deserved attention. In the first instance, our study was derived from retrospective cohort, which might easily introduce recall bias. The next, the suggested NLR cutoff is differ from previous study. Kawahara et al^[10]^ recommended 2.4 as the cutoff, but in present study, we found that 2.44 was a proper cutoff. We compared these 2 cutoffs (detailed in sTable 1). In entire cohort, the 2 different cutoffs showed a similar accuracy. But in the subgroup with PSA ranged from 4 to 10 ng.ml^−1^, positive and negative predictive values, using the 2.44 as cutoff were 46.8% and 83.9%, respectively, whereas, using the 2.4 as cutoff were 45.6% and 83.5%, respectively. Neutrophil and lymphocyte counts affected by various physiological, pathological, and physical factors, which may more or less influence the determining of the cutoff, though NLR was proved stability.^[9,45]^ What's more, we didn’t take body mass index, history of PCa, and some other known or unknown factors into consideration, which might somewhat bias the results in multivariate analyses. Furthermore, as previously mentioned, about 20% PCa may be misdiagnosed in the first prostate biopsy. In other words, some of patients diagnosed as benign in the biopsy are actually PCa patients, which may cover up the real relation. But, in our center, by taking biopsy of the prostate apex seriously, the comprehensive positive rate is about 26.36% among patients with PSA ranged 0 to 10 ng.ml^−1^, which might decrease the false-negative rate in a certain degree, making our data more solid and persuasive. Finally, we observed a promising result among patients with PSA ranged from 4 to 10 ng.ml^−1^. However, these analyses derived from a subgroup with limited sample size. Large-scaled prospective studies are needed to confirm our findings.

## 5 Conclusions

The patients with high-NLR value may have significant higher risk to be diagnosed with PCa, especially among the patients with PSA ranged from 4 to 10 ng.ml^−1^. In this subgroup, the adding of NLR value in the multivariate model can improve the accuracy of PCa prediction in a large degree. If validated, the NLR will become a promising, accessible, inexpensive biomarker for PCa prediction.

## Acknowledgments

The authors thank Lu Feng for manuscript revising.

## Supplementary Material

Supplemental Digital Content

## References

[1] Siegel RL Miller KD Jemal A Cancer statistics, 2016. *CA Cancer J Clin* 2016; 66:7–30. 26742998 10.3322/caac.21332

[2] Chen W Zheng R Baade PD Cancer statistics in China, 2015. *CA Cancer J Clin* 2016; 66:115–132. 26808342 10.3322/caac.21338

[3] Sundar S O’Cathail M Neutrophil-lymphocyte ratio is prognostic but not predictive of response to Abiraterone in metastatic castration-resistant prostate cancer. *JRSM Open* 2015; 6: 2054270415611332. 10.1177/2054270415611332 PMC4668914 26664732

[4] Yin X Xiao Y Li F Prognostic role of neutrophil-to-lymphocyte ratio in prostate cancer: a systematic review and meta-analysis. *Medicine (Baltimore)* 2016; 95:e2544. 26817900 10.1097/MD.0000000000002544 PMC4998274

[5] Gu X Gao X Li X Prognostic significance of neutrophil-to-lymphocyte ratio in prostate cancer: evidence from 16,266 patients. *Sci Rep* 2016; 6:22089. 26912340 10.1038/srep22089 PMC4766531

[6] Kawahara T Yokomizo Y Ito Y Pretreatment neutrophil-to-lymphocyte ratio predicts the prognosis in patients with metastatic prostate cancer. *BMC Cancer* 2016; 16:111. 26883640 10.1186/s12885-016-2134-3 PMC4754823

[7] Zhang GM Zhu Y Ma XC Pretreatment neutrophil-to-lymphocyte ratio: a predictor of advanced prostate cancer and biochemical recurrence in patients receiving radical prostatectomy. *Medicine (Baltimore)* 2015; 94:e1473. 26469891 10.1097/MD.0000000000001473 PMC4616804

[8] Yuksel OH Urkmez A Akan S Predictive value of the platelet-to-lymphocyte ratio in diagnosis of prostate cancer. *Asian Pac J Cancer Prev* 2015; 16:6407–6412. 26434851 10.7314/apjcp.2015.16.15.6407

[9] Oh JJ Kwon O Lee JK Association of the neutrophil-to-lymphocyte ratio and prostate cancer detection rates in patients via contemporary multi-core prostate biopsy. *Asian J Androl* 2015; 17:1–5. 10.4103/1008-682X.164198 PMC5109892 26470836

[10] Kawahara T Fukui S Sakamaki K Neutrophil-to-lymphocyte ratio predicts prostatic carcinoma in men undergoing needle biopsy. *Oncotarget* 2015; 6:32169–32176. 26359354 10.18632/oncotarget.5081 PMC4741667

[11] Gokce MI Hamidi N Suer E Evaluation of neutrophil-to-lymphocyte ratio prior to prostate biopsy to predict biopsy histology: results of 1836 patients. *Can Urol Assoc J* 2015; 9:E761–765. 26600880 10.5489/cuaj.3091 PMC4639422

[12] Kaynar M Yildirim ME Gul M Benign prostatic hyperplasia and prostate cancer differentiation via platelet to lymphocyte ratio. *Cancer Biomark* 2015; 15:317–323. 25586096 10.3233/CBM-150458 PMC12964681

[13] Luan Y Huang TB Gu X Effect of prostate volume on the peripheral nerve block anesthesia in the prostate biopsy: a strobe-compliant study. *Medicine (Baltimore)* 2016; 95:e4184. 27428215 10.1097/MD.0000000000004184 PMC4956809

[14] Templeton AJ McNamara MG Seruga B Prognostic role of neutrophil-to-lymphocyte ratio in solid tumors: a systematic review and meta-analysis. *J Natl Cancer Inst* 2014; 106:dju124. 24875653 10.1093/jnci/dju124

[15] Margolis KL Rodabough RJ Thomson CA Prospective study of leukocyte count as a predictor of incident breast, colorectal, endometrial, and lung cancer and mortality in postmenopausal women. *Arch Intern Med* 2007; 167:1837–1844. 17893304 10.1001/archinte.167.17.1837

[16] Iida M Ikeda F Ninomiya T White blood cell count and risk of gastric cancer incidence in a general Japanese population: the Hisayama study. *Am J Epidemiol* 2012; 175:504–510. 22366378 10.1093/aje/kwr345

[17] Guthrie GJ Charles KA Roxburgh CS The systemic inflammation-based neutrophil-lymphocyte ratio: experience in patients with cancer. *Crit Rev Oncol Hematol* 2013; 88:218–230. 23602134 10.1016/j.critrevonc.2013.03.010

[18] Heikkila K Ebrahim S Lawlor DA A systematic review of the association between circulating concentrations of C reactive protein and cancer. *J Epidemiol Community Health* 2007; 61:824–833. 17699539 10.1136/jech.2006.051292 PMC2703800

[19] Hamidi N Gokce MI Suer E Evaluation of increased preoperative serum high sensitive C-reactive protein and procalcitonin levels on grade and stage of clear cell renal cell carcinoma. *Clin Nephrol* 2015; 83:225–230. 25707457 10.5414/CN108448

[20] Ferro M De Cobelli O Buonerba C Modified glasgow prognostic score is associated with risk of recurrence in bladder cancer patients after radical cystectomy: a multicenter experience. *Medicine (Baltimore)* 2015; 94:e1861. 26496339 10.1097/MD.0000000000001861 PMC4620818

[21] Bruzzese D Mazzarella C Ferro M Prostate health index vs percent free prostate-specific antigen for prostate cancer detection in men with “gray” prostate-specific antigen levels at first biopsy: systematic review and meta-analysis. *Transl Res* 2014; 164:444–451. 25035153 10.1016/j.trsl.2014.06.006

[22] Beer TM Lalani AS Lee S C-reactive protein as a prognostic marker for men with androgen-independent prostate cancer: results from the ASCENT trial. *Cancer* 2008; 112:2377–2383. 18428198 10.1002/cncr.23461

[23] Toriola AT Laukkanen JA Kurl S Prediagnostic circulating markers of inflammation and risk of prostate cancer. *Int J Cancer* 2013; 133:2961–2967. 23754532 10.1002/ijc.28313

[24] Fujita K Imamura R Tanigawa G Low serum neutrophil count predicts a positive prostate biopsy. *Prostate Cancer Prostatic Dis* 2012; 15:386–390. 22777394 10.1038/pcan.2012.27

[25] Balkwill F Mantovani A Inflammation and cancer: back to Virchow? *Lancet* 2001; 357:539–545. 11229684 10.1016/S0140-6736(00)04046-0

[26] Jaiswal M LaRusso NF Burgart LJ Inflammatory cytokines induce DNA damage and inhibit DNA repair in cholangiocarcinoma cells by a nitric oxide-dependent mechanism. *Cancer Res* 2000; 60:184–190. 10646872

[27] O’Byrne KJ Dalgleish AG Browning MJ The relationship between angiogenesis and the immune response in carcinogenesis and the progression of malignant disease. *Eur J Cancer* 2000; 36:151–169. 10741273 10.1016/s0959-8049(99)00241-5

[28] Leek RD Landers RJ Harris AL Necrosis correlates with high vascular density and focal macrophage infiltration in invasive carcinoma of the breast. *Br J Cancer* 1999; 79:991–995. 10070902 10.1038/sj.bjc.6690158 PMC2362675

[29] Grivennikov SI Greten FR Karin M Immunity, inflammation, and cancer. *Cell* 2010; 140:883–899. 20303878 10.1016/j.cell.2010.01.025 PMC2866629

[30] Coussens LM Werb Z Inflammation and cancer. *Nature* 2002; 420:860–867. 12490959 10.1038/nature01322 PMC2803035

[31] Sutcliffe S Platz EA Inflammation in the etiology of prostate cancer: an epidemiologic perspective. *Urol Oncol* 2007; 25:242–249. 17483023 10.1016/j.urolonc.2006.09.014

[32] Sciarra A Mariotti G Salciccia S Prostate growth and inflammation. *J Steroid Biochem Mol Biol* 2008; 108:254–260. 17935971 10.1016/j.jsbmb.2007.09.013

[33] Nakai Y Nonomura N Inflammation and prostate carcinogenesis. *Int J Urol* 2013; 20:150–160. 22852773 10.1111/j.1442-2042.2012.03101.x

[34] Sfanos KS De Marzo AM Prostate cancer and inflammation: the evidence. *Histopathology* 2012; 60:199–215. 22212087 10.1111/j.1365-2559.2011.04033.x PMC4029103

[35] Taverna G Pedretti E Di Caro G Inflammation and prostate cancer: friends or foe? *Inflamm Res* 2015; 64:275–286. 25788425 10.1007/s00011-015-0812-2

[36] Lapi F Levi M Simonetti M Risk of prostate cancer in low-dose aspirin users: a retrospective cohort study. *Int J Cancer* 2016; 139:205–211. 26915905 10.1002/ijc.30061

[37] Veitonmaki T Murtola TJ Maattanen L Use of non-steroidal anti-inflammatory drugs and prostate cancer survival in the Finnish prostate cancer screening trial. *Prostate* 2015; 75:1394–1402. 26073992 10.1002/pros.23020

[38] Jacobs EJ Newton CC Stevens VL Daily aspirin use and prostate cancer-specific mortality in a large cohort of men with nonmetastatic prostate cancer. *J Clin Oncol* 2014; 32:3716–3722. 25332245 10.1200/JCO.2013.54.8875

[39] Huang TB Yan Y Guo ZF Aspirin use and the risk of prostate cancer: a meta-analysis of 24 epidemiologic studies. *Int Urol Nephrol* 2014; 46:1715–1728. 24687637 10.1007/s11255-014-0703-4

[40] Gupta S Srivastava M Ahmad N Over-expression of cyclooxygenase-2 in human prostate adenocarcinoma. *Prostate* 2000; 42:73–78. 10579801 10.1002/(sici)1097-0045(20000101)42:1<73::aid-pros9>3.0.co;2-g

[41] Kirschenbaum A Klausner AP Lee R Expression of cyclooxygenase-1 and cyclooxygenase-2 in the human prostate. *Urology* 2000; 56:671–676. 11018637 10.1016/s0090-4295(00)00674-9

[42] Nithipatikom K Isbell MA Lindholm PF Requirement of cyclooxygenase-2 expression and prostaglandins for human prostate cancer cell invasion. *Clin Exp Metastasis* 2002; 19:593–601. 12498388 10.1023/a:1020915914376

[43] Elwood PC Gallagher AM Duthie GG Aspirin, salicylates, and cancer. *Lancet* 2009; 373:1301–1309. 19328542 10.1016/S0140-6736(09)60243-9

[44] Zha S Yegnasubramanian V Nelson WG Cyclooxygenases in cancer: progress and perspective. *Cancer Lett* 2004; 215:1–20. 15374627 10.1016/j.canlet.2004.06.014

[45] Gabay C Kushner I Acute-phase proteins and other systemic responses to inflammation. *N Engl J Med* 1999; 340:448–454. 9971870 10.1056/NEJM199902113400607

