# Supplementary material for: Predictive value of neutrophil-to-lymphocyte ratio in diagnosis of prostate cancer among men who underwent template-guided prostate biopsy: A STROBE-compliant study
Source: Medicine (Baltimore). 2016 Nov 4;95(44):e5307. doi: 10.1097/MD.0000000000005307 (PMC5591156; doi:10.1097/MD.0000000000005307)
Supplement: Supplemental Digital Content [file medi-95-e5307-s001.docx]

sTable 1. Prediction of prostate cancer using two different NLR cutoffs (2.44 versus 2.4).

|  | **Sensitivity** | **Specificity** | **PPV** | **NPV** |
| --- | --- | --- | --- | --- |
| Entire cohort |  |  |  |  |
| NLR>=2.44 | 55.2% | 52.8% | 51.8% | 56.2% |
| NLR>=2.4 | 56.5% | 51.0% | 51.4% | 56.1% |
| PSA: 4-10 ng/ml |  |  |  |  |
| NLR>=2.44 | 72.0% | 64.0% | 46.8% | 83.9% |
| NLR>=2.4 | 72.0% | 62.3% | 45.6% | 83.5% |

PPV: positive predictive value, NPV: negative predictive value
